# Mycetoma: Nonvenereal perineal lesions

**DOI:** 10.4103/2589-0557.69000

**Published:** 2010

**Authors:** Shweta Gupta, Khushbu Jain, Chirag Parmar, Parul Shah, Ranjan C. Raval

**Affiliations:** Department of Skin and VD, V.S. General Hospital, Ahmedabad 380 006, Gujarat, India; 1 Department of Microbiology, V.S. General Hospital, Ahmedabad 380 006, Gujarat, India

**Keywords:** Eumycetoma, melanoid, mycetoma

## Abstract

Mycetoma is a chronic, granulomatous disease of the skin, and subcutaneous tissue, which sometimes involves muscle, bones, and neighboring organs. It is characterized by tumefaction, abscess formation, and fistulae with discharge of grains from sinuses. Mycetoma can be caused by various species of fungi (eumycetoma) and aerobic actinomycetes (actinomycetoma), which occur as saprophytes in soil or plants. A tentative diagnosis sufficient to initiate treatment may be made on the basis of grain color. For instance, melanoid grains are always caused by fungi and ochroid or pale grains by actinomycetes. Although this is not the thumbrule, there are exceptional reports too. As trauma favors infection, most lesions are on the foot and lower leg but they may occur anywhere on the body mimicking actinomycosis. However, lab investigations and culture are important tool to differentiate apart from the clinical picture. We are reporting atypical case with unusual site of presentation (perineum and thigh) of mycetoma.

## INTRODUCTION

Mycetoma is a Greek term for “fungal tumor.” It is a granulomatous infection of dermal and subcutaneous tissue that may extend to muscle or even bone and is due to implantation. Mycetoma is differentiated from other mycoses by its characteristic draining sinuses containing grains (sclerotic, sulfure granules) and local edema. Three different subtypes exist: actinomycotic mycetoma (caused by filamentous aerobic and anaerobic organism, *Nocardia brasiliensis, Actinomadura madurae*), eumycotic mycetoma (caused by true fungi), and Botryomycosis (caused by true bacteria, e.g., *Staphylococcus aureus, Pseudomonas*). These occur as saprophytes in soil or plants.[1] From these sources, they are implanted subcutaneously, usually after penetrating injury.[2,3] In India, actinomycotic mycetoma is more commonly encountered than eumycotic mycetoma.[4]

The earliest stage is a firm painless nodule but, with time, papules, pustules that break down to form sinuses, appear on the skin surface. The whole area becomes hard and swollen often without significant pain. Individuals who are affected seek medical attention mainly because of the tumefaction and draining sinuses. Lymph node involvement is rare.[5] The diagnosis is made by clinical presentation, type of granules, KOH, Biopsy, and microbiological culture. Black grains are always caused by fungi and red grains by actinomycete.[6] White or pale grains may be either eumycotic or actinomycotic.[7] Radiologic tests (bone radiographs) should be performed if underlying bony involvement is suspected. MRI provides the most comprehensive method.[8] Several immunologic assays using culture filtrate or cytoplasmic antigens of mycetoma agents have been developed to detect antibodies. For the best outcome, eumycotic mycetoma must be diagnosed early and surgically excised before the underlying bone become involved. Once the lesion has been excised, systemic antifungal therapy is administered. The medical treatment of actinomycotic mycetoma consists primarily of streptomycin or amikacin plus either trimethoprim-sulfamethoxazole, or dapsone; therapy is continued for months to years.

## CASE REPORT

**Figure 1:**
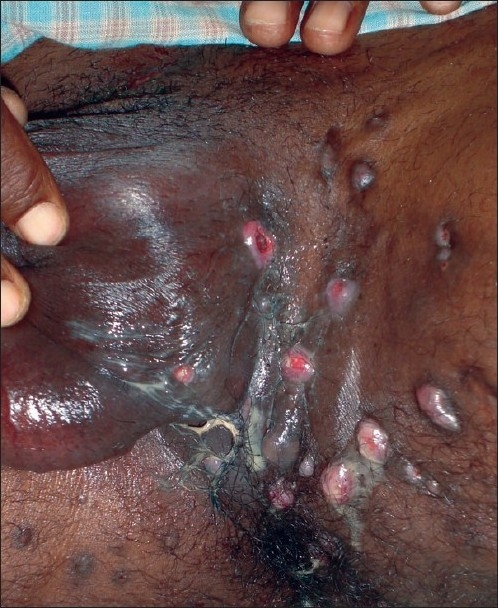
Perineal lesions

**Figure 2:**
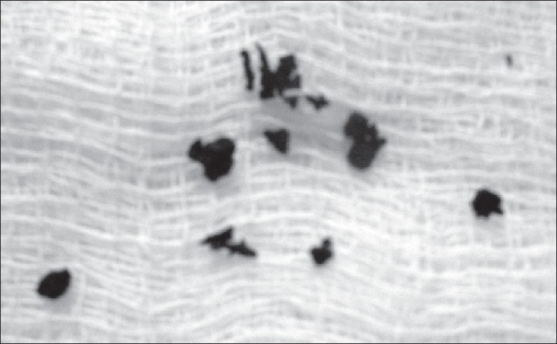
Melanotic granules expressed out from the lesions

A 36-year-old man, farmer by occupation, presented with 8-year history of nonhealing lesions on the thigh and genitals [Figure 1]. The condition started as a single painless nodule followed by development of multiple brownish papules and nodules in the surrounding area in about a year. The patient was otherwise healthy with negative surgical or medical history of any long-term illness other than this one. There was no history of any trauma at the site. On examination, there were many discharging sinuses simulating “water can” appearance of Lymphogranuloma venereum (LGV) discharging pus. On pressing, the dark melanotic granules were expressed out [Figure 2]. All routine investigations were performed along with biopsy, KOH mount of the granules, and the X-ray of the local part to judge the extent of the lesions. Chest X-ray was normal. MT was negative. S. HIV and S.VDRL were normal. Tentative diagnosis of deep granuloma was made.

**Figure 3:**
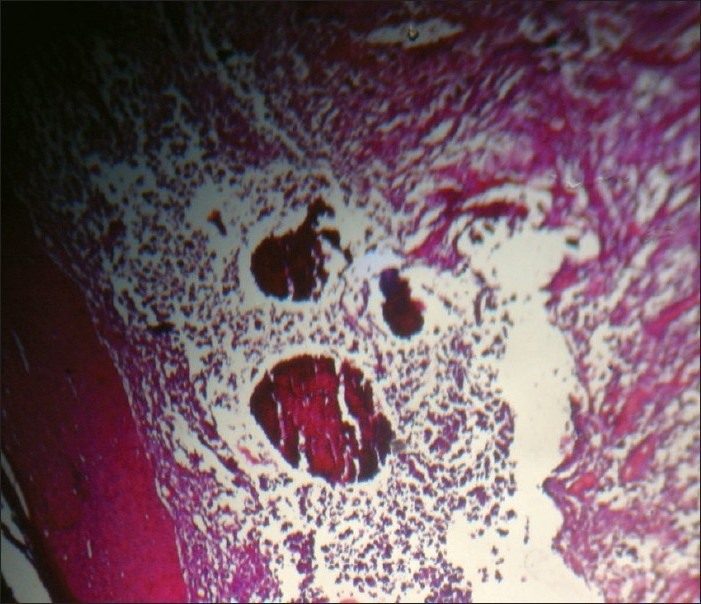
H and E histopathology with organized aggregates of filaments

**Figure 4:**
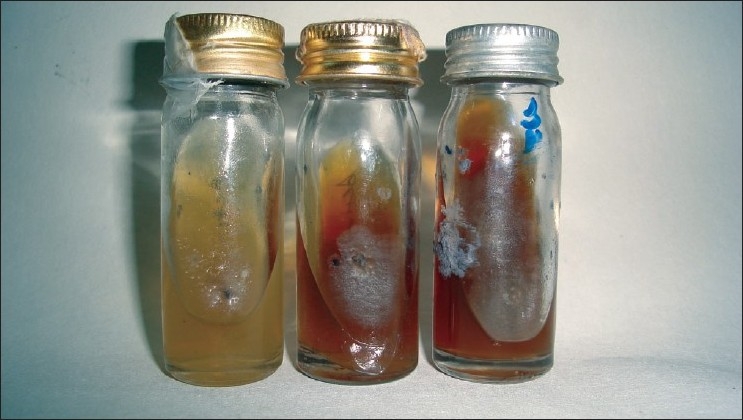
*Madurella mycetomatis* in Sabouraud’s dextrose agar (two cultured specimens of patient) with leathery colony and brown pigment production compared with control

The KOH mount of the granule revealed thin long-branched hyphae with well-defined walls. The punch biopsy of the nodule on histopathological examination revealed dermis with mixed inflammatory cell infilterate mainly of the polymorphs and lymphocytes. There were neutrophilic abcesses with organized aggregates of filaments [Figure 3] and 1% AFB revealed no acid fast filaments. On Gram stain of the granule, fungal elements were present in the granules. On culture of the granules (washed with sterile saline and inoculated on to Sabouraud’s dextrose agar) revealed the organism to be *Madurella mycetomatis* [Figure 4].

With the confirmation of diagnosis patient was continued on T. Itraconazole (100) 2 tab. BD, T. Dapsone (100) 1 tab. OD with regular follow-up. After 1 month, Tab. Dapsone was stopped on follow-up. The frequency and number of black granules decreased within 2 months and lesions started to heal with very little pus discharge. After 4 month of the therapy with Itraconazole, patient was referred to surgery department for debridement. The local area was excised fully with repeat histopathological examination of the excised specimen and skin graft at the excised site and patient was continued with Ketoconazole for 1 month.

## DISCUSSION

Mycetoma is usually found on lower legs and foot due to more chances of injury but can be found at unusual sites such as perineum and genitals mimicking many venereal diseases. Mycetoma should be differentiated from actinomycosis, botryomycosis, osteomyelitis, neoplasms, Kaposi’s sarcoma, syphilis, yaws, leprosy, tuberculosis, cutaneous leishmaniasis, and other mycosis. Negative Montoux test (MT), normal routine investigations, nonreactive S.HIV and S. VDRL, no H/o any genital mucosal lesions in the past. Negative sexual history excluded the other differential diagnosis. The melanoid granules from the lesions narrowed the differential diagnosis (mycetoma, actinomycosis, and Botryomycosis.) further to presumptive of mycetoma (eumycetoma). It was presumptive diagnosis because few actinomycetoma are reported to have melanoid granules also. Histologic examination not only establishes the diagnosis, but also allows specific identification of the causal agent in 94% of cases.[9] Splendore-Hoeppli reaction usually denotes the actinomycetoma with few exception cases of eumycetoma. Culture confirmed the diagnosis.

## CONCLUSION

Every genital lesion is not venereal in its origin. The patient should be properly evaluated clinically and by laboratory investigations so that proper treatment can be started in time preventing the dreadful sequels. The color of grains help a great deal to make a presumptive diagnosis so that proper treatment of mycetoma can be started in time depending on whether it is actinomycetoma or eumycetoma. The surgical debridement makes one of the important line of management in mycetoma along with medical treatment consisting of T. Itraconazole 400 mg for 4 months and if needed then administration of T. Ketoconazole 400 mg for 1–2 months more. LFT and routine investigations should be performed on monthly follow-ups.
